# Gram negative periprosthetic hip infection: nearly 25% same pathogen infection persistence at a mean of 2 years

**DOI:** 10.1007/s00402-023-05104-5

**Published:** 2023-11-01

**Authors:** Daniel Karczewski, Johann Scholz, Christian Hipfl, Doruk Akgün, Marcos R. Gonzalez, Sebastian Hardt

**Affiliations:** 1 Department of Orthopaedic Surgery and Traumatology, Charité Berlin, University Hospital, Chariteplatz 1, 10117 Berlin, Germany; 2 Department of Orthopaedic Surgery, Musculoskeletal Oncology Service, Massachusetts General Hospital, Harvard Medical School, 55 Fruit Street, Boston, MA 02114 USA

**Keywords:** Relapse, Infection persistence, Hip revision, Difficult to treat pathogens, *E. coli*

## Abstract

**Purpose:**

While gram negative (GN) periprosthetic joint infections (PJI) have previously been described as difficult to treat pathogens with high rates of reinfection, limited investigations have addressed midterm outcomes and risk of infection persistence by the same pathogen. This study analyzed (1) baseline demographics, treatment strategy, and midterm outcomes of GN PJIs, as well as (2) differences in reinfection and relapse rates compared to gram positive (GP) PJIs.

**Methods:**

We identified 29 patients that were revised for 30 GN PJIs of total hip arthroplasties (THAs) between 2010 and 2020 using a university-based hip registry. Mean age was 77 years, 63% were females (19), and mean BMI was 27 kg/m^2^. Major causative pathogens included *Escherichia coli* (12), *Klebsiella pneumoniae* (5), *Pseudomonas aeruginosa* (5), and *Enterobacter cloacae* complex (5). Mean follow-up was 3.5 years. Study outcomes included (1) Kaplan–Meier survivorship analyses of all 30 GN PJIs, and (2) comparison of 18 two-stage exchanges for GN PJIs and 104 two-stage exchanges for GP PJIs, performed during the time from 2013 to 2017.

**Results:**

(1) The 5-year survivorship free of recurrent PJI was 69%, and there were 7 recurrent PJIs at a mean of 2 years. There were 2 further suprafascial wound infections, resulting in a 61% survivorship free of any infection at 5-years. At a mean of 2 years, there were 7 patients with reinfection by the same GN pathogen (6 PJIs, one wound infection) as at index revision (23%). (2) Following two-stage exchange, the 5-year survivorship free of recurrent PJI (GN: 74%; GP: 91%; p = 0.072), any infection (GN: 61%; GP: 91%; p = 0.001), and reinfection by the same pathogen was significantly lower among GN PJIs (GN: 73%; GP: 98%; p < 0.001).

**Conclusions:**

Patients revised for GN PJIs are at increased risk of reinfection as opposed to GP infections. Affected patients must be counseled on the exceptionally high risk of infection persistence with one in four developing relapses.

**Level of evidence:**

Therapeutic Level III.

## Introduction

Outcomes of periprosthetic joint infections (PJIs) depend on a number of factors, including age, secondary diseases, previous revisions, soft tissue conditions, and treatment strategy [1, 2]. In addition to patient dependent and surgical risk factors, the involved pathogen is known to impact reinfection rates and long-term chances of cure [3]. Among Candida and rifampin resistant gram positive (GP) bacteria, PJIs caused by gram negatives (GN) have historically been associated with poor outcome [4–6].

Although GN bacteria are considered an atypical cause of PJI, they still represent an estimated 5 to 15% of all cases [1, 7]. Moreover, their prevalence is even further increased if affecting total hip arthroplasties (THAs) compared to total knee arthroplasties (Tande et al. Mayo Clinic PJI database) [1]. Despite increasing antibiotic multi resistance among GNs, an overall increase in numbers, and affecting nearly one in 10 prosthetic hip infections, only a handful of studies have addressed GN as causative agent for PJI [8, 9]. Moreover, did the limited studies on GN PJIs focus on a specific type of surgery only [10, 11], were primarily short-term reports [7, 12], or did not analyze reinfection characteristics and risk of infection persistence by the same pathogen in many cases [7, 12].

As such, this single university-based investigation analyzed GN PJIs in THAs at midterm outcome. We aimed to characterize baseline demographics, infection characteristics, and surgical strategies. Moreover, did this study determine the risk of infection persistence by the same GN pathogen, and analyzed differences between GP and GN PJIs with respect to reinfection and infection persistence rates.

## Patients and methods

### Study design

After obtaining institutional review board approval, we identified 29 patients that were revised for 30 GN prosthetic hip infections between 2010 and 2020. Mean age was 77 years (range, 56 to 89), mean BMI was 27 kg/m^2^ (range, 18 to 41 kg/m^2^), and 63% were females (19). Mean American Society of Anaesthesiologists (ASA) [13] score was 3 (range, 1 to 3). Five patients had diabetes mellitus, 3 rheumatoid arthritis, and 4 chronic obstructive pulmonary disease and hypothyroidism each. Mean follow-up was 3.5 years (range, 1 month to 7 years).

### Pathogens

All 30 PJIs were confirmed infections according to the 2021 European Bone and Joint Infection Society (EBJIS) criteria [14]. GN pathogens included *Escherichia coli* (12), *Klebsiella pneumoniae* (5), *Pseudomonas aeruginosa* (5), and *Enterobacter cloacae* complex (5), *Proteus mirabilis* (2), *Morganella morganii* (2), *Acinetobacter baumannii* (2), *Finegoldia magna* (2), and *Corynebacterium tuberculostearicum* (1). Both patients affected by *Finegoldia magna* were simultaneously affected by *Escherichia coli* and *Enterobacter cloacae* complex, respectively. Seventeen of the 30 GN PJIs had a coexisting GP pathogen at time of revision (57%), including *coagulase negative Staphylococci* (CNS; 10), *Staphylococcus aureus* (4), *Enterococcus faecalis* (3), and one case of a mixed polymicrobial infection (*Propionibacterium acnes*, *Enterococcus faecalis*, *Staphylococcus aureus*, CNS) (Table 1). As such, 12 GN PJIs were monomicrobial (40%). Pathogens, for which no biofilm-active antibiotics were available were considered difficult-to-treat (DDT) pathogens [15]. In this series, those included rifampin-resistant Staphylococci (1), fluoroquinolone-resistant GN bacteria (6), and Enterococci (2). High-virulence pathogens were defined according Zimmerli et al. [16] and included *Staphylococcus aureus*, Streptococci and Enterococci. In our cohort, high-virulence bacteria were involved in 7 cases.

### Infections

Infection type (I in 6, II in 4, and III in 20 patients), systemic host grade (A in 5, B in 17, C in 8 cases), and local risk factors (1 in 6, 2 in 16, 3 in 8 joints) were recorded based on the McPherson classification [17]. Among the 30 PJIs, there was one synchronous and one metachronous infection of other prosthetic joints. In total, 5 patients had an additional joint prosthesis (THAs or TKAs) at time of GN PJI. The only synchronous PJI in this cohort affected a patient with simultaneous infections of both of his THAs. There was one case of a metachronous infection of a contralateral THA with subsequent two-stage exchange one year prior to the GN PJI in this cohort. Twenty-six joints were revised prior to the current intervention (mean 3, range 1 to 8), including 21 for PJI (mean 2, range 1 to 5). Mean C-reactive-Protein (CRP) was 83 mg/l (range, 2 to 348 mg/l). Seven patients had a fistula at initial presentation (23%), 5 additional patients an abscess (17%). Fifteen THAs were cemented (50%), including 7 hybrid cementations (23%). Signs of loosening were noted in 15 acetabular and femoral components each (50%). Ten patients had loosening of both components prior to revision (33%).

### Surgical procedures

For all patients with suspected PJI, our diagnostic algorithm included preoperative aspirations, intraoperative collection of at least five tissue samples of different localization, as well as sonication of explanted material and intraoperative aspirations, if possible [18]. In our center, an acute PJI with adequate bone and soft tissue quality, a fixed prosthesis, and no involvement of DDT pathogens was addressed by debridement and preservation of the prosthesis with surgical exchange of the mobile components (DAIR). In the case of chronic PJI with a symptom onset of more than 4 weeks, complete prosthesis removal was performed. Thereby a two-stage exchange was used in the presence of DTT pathogens, fistulae or multiple prior revisions, as well as in culture negative infections. We considered long-term antibiotic suppression in the setting of an unsatisfactory outcome, when eradication of the infection was not possible [19]. Two-stage exchange was performed in 18, one-stage exchange and debridement, antibiotics and implant retention with exchange of mobile parts (DAIR) in 5 cases each, whereas permanent resection arthroplasty was necessary in 2 patients (Table 1). In case of two-stage exchanges, mean time between resection arthroplasty and reimplantation was 10 weeks (range, 4 to 25 weeks), and interim debridement performed in 9 cases (50%). In 4 of the 9 cases, the same GN pathogen was identified compared to resection arthroplasty (3-times *Escherichia coli*, one-time *Proteus mirabilis*). Mean time required for implant removal and reimplantation were 139 min (range, 29 to 265 min) and 177 min (range, 84 to 299 min), respectively.

### Statistical analysis

Study endpoints included survivorship free of PJI, any infection, and reinfection by the same pathogen (relapse). The 2021 EBJIS criteria were used to define recurrent PJI [14], whereas any infection was considered any PJI and any additional supra fascial wound infection with identification of a pathogen (as opposed to simple wound healing delay). Secondary endpoints included a comparison of survivorship between two-stage exchanges performed for GN as opposed to GP PJIs. Survivorship analysis was based on Kaplan Meier curves [20]. Differences in survivorship were calculated using a log-rank test, differences in continuous variables with a t- and Whitney-U-man test, and differences between categorial variables via a fisher-exact test. SAS version 9.4 (SAS Institute, Inc., Cary, North Carolina) was used for calculations.

## Results

### Outcome

The 5-year survivorship free of death was 76% (95% CI 56 to 96%; 10 patients at risk). Five patients died at a mean of 2 years (range, 6 days to 5 years). One patient died by perioperative non PJI related complications. Likewise, none of the other 4 patients died by PJI related complications. The 5-year survivorship free of any recurrent PJI was 69% (95% CI 50 to 89%; 14 patients at risk), and there were 7 recurrent PJIs at a mean of 2 years (range, 16 days to 4 years). There were 2 further suprafascial wound infection with identification of pathogens at 2 months (*Finegoldia magna*) and 5 months (*Bacillus cereus*, *Propionibacterium acnes*), resulting in a 5-year survivorship free of any infection of 61% (95% CI 40 to 81%; 12 patients at risk). At a mean of 2 years, there were 7 patients (6 PJIs, one wound infection) with reinfection by the same GN pathogen as at index revision (23%). Cases of reinfection by the same pathogen involved *Escherichia coli* (3), *Pseudomonas aeruginosa* (2), *Enterobacter cloacae* (1) and *Proteus mirabilis* (1). In addition to the above-mentioned infection related revisions, there was one liner exchange for dislocation at one month. There were 2 further non-operative complications, and both were closed reductions for dislocations at one and 3 months (Table 1). Mean preoperative Harris Hip Score [21] was 29 (range, 12 to 48) and increased to 60 (range, 19 to 70) at last follow-up.

**Table 1:** Characteristics of patients revised for GN PJI

THA	Age (years)	Sex	Pathogens	Surgical procedure	Outcome
1	73,4	Female	*E. coli*	Two-stage exchange	No complication
2	79,1	Male	*E. coli*, *Staph. epidermidis*, *Staph. aureus*	Two-stage exchange	Suprafascial wound infection
3	80,1	Male	*E. coli*, *Staph. epidermidis*	Two-stage exchange	No complication
4	79,4	Female	*E. coli*	Two-stage exchange	Recurrent PJI, reinfection by same pathogen
5	57,0	Female	*Enterobacter cloacae*, *Staph. epidermidis*	Two-stage exchange	No complication
6	80,1	Female	*E. coli*, *Enterobacter cloacae*, *Finegoldia magna*	Two-stage exchange	Suprafascial wound infection and closed reduction after dislocation
7	74,8	Male	*E. coli*	Two-stage exchange	No complication
8	78,8	Female	*Pseudomonas aeruginosa*, *Staph. epidermidis*	Two-stage exchange	Liner exchange for dislocation
9	55,6	Female	*Enterobacter cloacae*, *Staph. aureus*	Two-stage exchange	No complication
10	76,0	Female	*Klebsiella pneumoniae*, *Staph. epidermidis*	Two-stage exchange	No complication
11	83,3	Female	*E. coli*	Two-stage exchange	No complication
12	76,5	Female	*E. coli*, *Finegoldia magna*, *Enterobacter cloacae*	Two-stage exchange	No complication
13	71,0	Female	*Proteus mirabilis*, *Enterococcus faecalis*, *Staph. aureus*, *Staph. capitis*, *Staph. epidermitdis*, *Proprionibacterium acnes*	Two-stage exchange	No complication
14	82,8	Male	*Morganella morganii*, *Enterococcus faecalis*	Two-stage exchange	No complication
15	73,0	Female	*Pseudomonas aeruginosa*, *Staph. aureus*	Two-stage exchange	Recurrent PJI, reinfection by same pathogen
16	82,0	Male	*Morganella morganii*, *Pseudomonas aeruginosa*, *Enterococcus faecalis*	Two-stage exchange	No complication
17	79,0	Female	*E. coli*	Two-stage exchange	Recurrent PJI, reinfection by same pathogen
18	76,2	Male	*Enterobacter cloacae*	Two-stage exchange	Recurrent PJI, reinfection by same pathogen
19	87,3	Female	*E. coli*, *Staph. hominis*, *Staph. epidermidis*	One-stage exchange	Recurrent PJI, reinfection by same pathogen
20	87,2	Female	*E. coli*	One-stage exchange	No complication
21	71,2	Male	*Klebsiella pneumoniae*, *Corynebacterium tuberculostearicum*	One-stage exchange	No complication
22	60,7	Female	*Klebsiella pneumoniae*, *Staph. epidermidis*	One-stage exchange	No complication
23	89,1	Female	*Acinetobacter baumannii*, *Enterococcus casseliflavus*, *Staph. epidermidis*	One-stage exchange	No complication
24	89,0	Female	*Klebsiella pneumoniae*	DAIR	Closed reduction after dislocation
25	87,0	Female	*Pseudomonas aeruginosa*	DAIR	No complication
26	73,7	Male	*Proteus mirabilis*	DAIR	Recurrent PJI, reinfection by same pathogen
27	77,1	Female	*E. coli*	DAIR	No complication
28	71,8	Male	*Klebsiella pneumoniae*	DAIR	No complication
29	76,2	Male	*Pseudomonas aeruginosa*, *Propionibacterium acnes*	Resection arthroplasty	Wound infection, reinfection by same pathogen
30	76,4	Male	*Acinetobacter baumannii*, *Staph. epidermidis*	Resection arthroplasty	No complication

### GN versus GP PJI

**Fig. 1:**
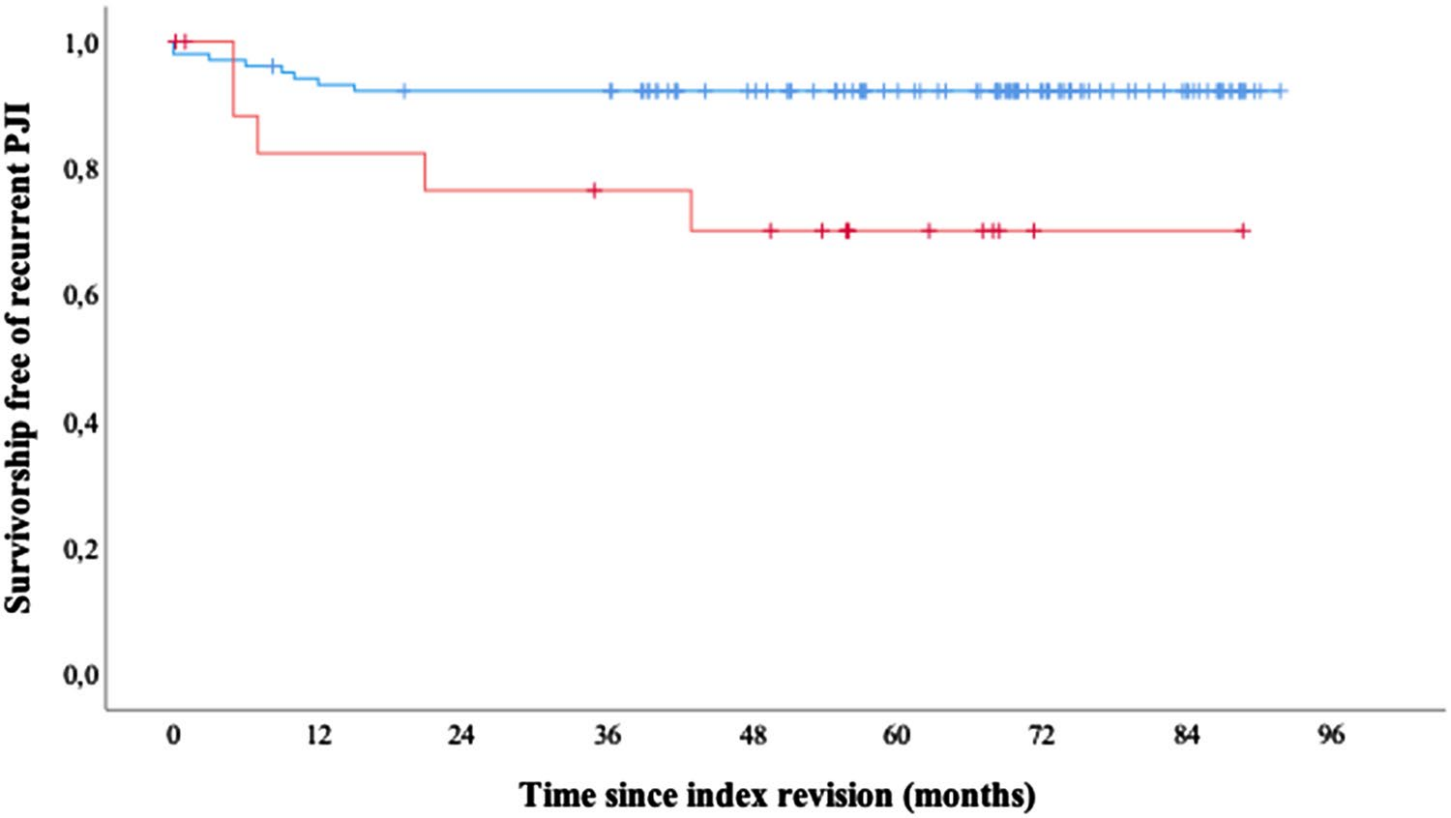
Kaplan–Meier survival curves of 18 GN and 104 GP two-stage THA exchange revisions. Curves with censored data (vertical spikes) are shown for the two cohorts (red graph: GN PJI, blue graph: GP PJIs). Five-year survivorship free of recurrent PJI was 74% (95% CI 58.9 to 81.3) and 91% (95% CI 89.5 to 94.9), respectively

A total of 18 patients underwent two-stage exchange for GN PJI in this cohort. Two-stage exchanges was used as a comparison group, representing the most commonly used treatment type in our clinic. During the same time period, between 2013 and 2017, there were 104 patients that were revised with a two-stage exchange for GP PJI, 8 of which had a recurrent PJI. Culture negative PJIs were excluded for comparison. Except a statistically significantly higher age among patients treated for GN PJIs, both groups did not differ in baseline demographics (Table 2). Following two-stage exchange, the 5-year survivorship free of recurrent PJI (GN: 74%, GP: 91%; p = 0.072) (Fig. 1), any infection (GN: 61%, GP: 91%; p = 0.001), and reinfection by the same pathogen were all significantly lower among GN PJIs (GN: 73%, GP: 98%; p < 0.001).

**Table 2:** Comparison between patients revised with two-stage exchange for GN and GP PJIs

	Gram negative PJIs	Gram positive PJIs	P
Patients (n)*	18	104	–
Age (years)^**†**^	75 ± 8	70 ± 9	0.014
Females (n)*	12 (67)	58 (56)	0.448
Prior revision for PJI (n)*	13 (72)	56 (54)	0.199
BMI (kg/m^2^)^**†**^	27.0 ± 6	29 ± 6	0.256
ASA^**†**^	3 ± 0.5	3 ± 0.6	0.254
Polymicrobial infections (n)*	7 (39)	36 (35)	0.792
McPherson infection type (n)*			
I	3 (17)	6 (6)	0.133
II	4 (22)	14 (13)	
III	11 (61)	84 (81)	
McPherson host grade (n)*			
A	2 (11)	18 (17)	0.776
B	11 (61)	62 (60)	
C	5 (28)	24 (23)	
McPherson local status (n)*			
1	3 (17)	5 (5)	0.074
2	10 (55)	81 (78)	
3	5 (28)	18 (17)	
CRP (mg/l)^**†**^	83 ± 94	49 ± 72	0.153
Interim length (weeks)^**†**^	10 ± 5	10 ± 10	0.911

*Results reported as absolute numbers and percentages, n (%)

^†^Results reported as means and standard deviation

## Discussion

While the number of PJIs will increase significantly over the next decades [22, 23], limited remains known on GN PJIs. As such, we analyzed 29 patients affected by 30 GN PJIs at a mean of 3.5 years follow-up. We found nearly one in 4 patients to experience infection persistence by the same pathogen at a mean of 2 years. In addition, we identified the rate of infection persistence to be significantly higher compared to GP cases at midterm follow-up.

Knowledge on baseline demographics of patients affected by periprosthetic hip infection is important, as certain pathogens are attributable to specific risk populations [24]. We found GN PJIs to primarily affect elderly, multimorbid, and overweight patients. These factors are also reflected by a poor local and systemic McPherson grade. In fact, two in three joints were revised for PJI in the past. This also confirms findings of Akkaya et al. that identified a significantly increased rate of DTT pathogens, including GN infections, in 66 patients with failed one-stage exchange of the knee [25]. Importantly, however, a comparison with GP PJIs revealed no statistically significant difference, except a higher age among GN cases. Of note, a higher age among GN PJIs falls in line with one previous report [7], although other studies could not confirm this finding [5, 26]. While prelim findings indicate similar baseline demographics between GN and GP PJIs, larger population samples will be needed to determine potential epidemiological differences in the future [5, 7, 26].

The leading GN pathogen in this cohort was *Escherichia coli* (40%), reflecting most previous studies on GN PJIs [5, 9]. Importantly, this investigation found a high percentage of polymicrobial and mixed GP-GN infections. In fact, only 40% of cases were monomicrobial. This rate is substantially lower than previously reported and might represent the fact that this investigation had a 70% proportion of patients that were revised for PJI in the past, as opposed to previous studies focusing on first time PJIs only [7]. We believe the inclusion of mixed GN-GP infections to be important, as it more accurately reflects the majority of patients encountered in clinic.

Outcomes of GN PJIs are known to be poor, with survivorship rates free of recurrent PJI to be reported as low as 27% at 2 years for implant retention attempts [7]. Importantly, the majority of previous studies did report of short-term outcomes only [5, 7, 9]. In contrast, this investigation analyzed midterm outcomes and found a 69% survivorship free of PJI recurrence at 5 years. This falls in line with the 5-year outcomes of 2 studies on DAIR for GN PJIs, although treatment failure was defined broader in both investigations [12, 27].

Similar to one previous investigation, we found the rate of reinfection to be significantly higher among GN as opposed to GP PJIs [7]. Importantly, we performed a detailed follow-up on the risk of infection persistence, and found nearly one in 4 patients to develop infection relapse by the same GN pathogen at a mean of 2 years. A similarly high rate of relapse was also reported by Martínez-Pastor et al. [10] with 8 of 35 patients experiencing relapse with the same GN pathogen at a median follow-up of 463 days. Uniquely, this investigation found relapse rates to be significantly increased compared to the GP group. This finding is important, as it demonstrates a unique feature of GN PJIs: patients are at risk of infection relapse rather than new infection by a different pathogen.

We acknowledge limitations to this study. First of all, did we present a single center cohort study with a subsequent low patient number in the light of an overall rare condition. Moreover, were treatment approaches and patient characteristics heterogenous, reducing comparability with existing studies. Finally, did this study not report GN PJIs in isolation, but rather included a high proportion of mixed and polymicrobial infections, as encountered in the daily clinical routine. This finding possibly impacts prognosis [28], although the polymicrobial infection rate were comparable to GP pathogens in the course of a two-stage exchange sub-analysis.

In conclusion, this investigation found GN PJIs to affect high-risk cohorts with the majority of patients being revised for PJI in the past. GN rarely occurred in isolation, but present with a high rate of polymicrobial infections (60%). Patients should be counseled on the increased risk of reinfection compared to GP PJIs, as well as a high chance of infection persistence by the same pathogen (23%). Based on our findings, we recommend prolonged suppression therapy of at least three months if involving GN pathogens. Future studies should focus on long-term outcomes, as well as multi center-based cohorts to increase the number of patients in a rare but difficult to treat condition.
